# Evaluation of the clinical success of four different types of lithium disilicate ceramic restorations: a retrospective study

**DOI:** 10.1186/s12903-021-01987-1

**Published:** 2021-12-07

**Authors:** Sharo Abdulrahman, Constantin Von See Mahm, Ranjdar Talabani, Darwn Abdulateef

**Affiliations:** 1 Department of Medicine and Dental Medicine, Danube Private University, Steiner Landstraße 124, 3500 Krems-Stein, Austria; 2 Conservative Department, College of Dentistry, University of Sulaimani, Madam Mitterrand Street 30, Sulaimani, Kurdistan Region 46001 Iraq

**Keywords:** Lithium disilicate, Clinical outcomes, Failure rate

## Abstract

**Background/purpose:**

How long do lithium disilicate restorations last before they fail? The aim of this study was to assess the success rate of four different types of restorations made from lithium disilicate.

**Materials and methods:**

A total of 87,203 ceramic restorations, classified into four different types (inlay or onlay, veneers (Vs), single crowns (SCs), and fixed partial dentures (FPDs)), were used. All were made of lithium disilicate (IPS e.Max CAD) with Cerec Inlab CAD/CAM system (Sirona Dental Systems, Bensheim, Germany). They were reported by dentists and entered in the database of the private B&R Dental Center between March 2015 and June 2020 and assessed retrospectively up to a period of 5 years based on the following parameters: failure rate and cause of failures (ceramic fracture, debonding, marginal adaptation, color match, endodontic intervention, periodontal disease, and secondary caries). Failure distribution according to gender, arch, and teeth type was also evaluated. The time-dependent time-to-failure/complication and their differences were calculated in months according to the Kaplan Meier and log-rank tests. The Chi-squared test (p 0.05) was used to assess the variations in causes of failure rates between different restorations.

**Results:**

Kaplan Meier test showed overall cumulative survival probability of lithium disilicate restorations for up to years was 85.08%. Inlay/onlay and Vs ceramic restorations showed highest cumulative survival probability (99.4%, 98.6, respectively). FPDs had the least cumulative survival probability (52.9%) which was significantly (P < 0.00001) higher than for other ceramic restorations using the log-rank test. Moreover, overall time-dependent time-to-failure/complication occurred after 52.373 months according to Kaplan–Meier (CI: lower bound: 51.875 months; upper bound: 52.871 months). Ceramic fracture in both FPDs and SCs (27.6% and 26.6%, respectively) and debonding in Vs (12.7%) were significant as the main reasons for failure (P = 0.000). The failure rate was significantly higher for the maxillary arch than the mandibular arch (P = 0.021). Fracture and marginal discrepancy were more frequent in the molar region (77.5% and 14.75%, respectively) and significantly higher here than in the anterior and premolar regions (P = 0.000).

**Conclusion:**

The medium-term performance of lithium disilicate is ideal. Ceramic fracture was the most common cause of failure in SCs and FPDs. FPDs presented with the highest failure rate based on evaluation for up to 5 years.

## Introduction

Lithium disilicate ceramic was first introduced to the market in 1998, under the name IPS Empress 2 (IvoclarVivadent, Schaan, Principality of Liechtenstein), for use with press technology. According to Tysowsky [1], the material generally consists of “70% needle-like lithium disilicate crystals which are embedded in a glassy matrix”. In 2005, IPS Empress 2 was replaced by a modified version, IPS e.max Press and IPS e.max CAD. The CAD version of the IPS lithium disilicate e.Max ceramic is provided in a metasilicate state, which is characterized by 40% platelet-shaped lithium metasilicate crystals and a glassy matrix, and is bluish in color. The material can be easily machined and try-in procedures can be accomplished with care. To obtain the lithium disilicate structure, the crystallization method is required. The final material properties and a tooth colored shade are obtained by a crystallization firing at 840 °C, which takes approximately 25 min [2].

In conservative dentistry, lithium disilicate has a number of advantages. First, it combines high mechanical strength of up to 360 ± 60 MPa with fracture toughness of between 2.0 and 2.5 MPa × m^0.5^ [2, 3]; second, it has translucent characteristics appropriate for tooth-colored restorations [4, 5]; third, with chair-side CAD/CAM technology only a single visit appointment is required, with no provisional phase [6]; fourth, the fresh ground dentin provides the best adhesive bond [7]; fifth, an immediate evaluation of the preparation and the margin is possible based on the digital impression procedure [8].

Lithium-disilicate has applications in a comprehensive range of products for diverse uses and processing techniques [9]. It is a glass–ceramic material that has the benefit of providing maximum esthetics [10] and good fracture resistance [11]. Based on these benefits, lithium disilicate could be implemented as a veneering material, for inlays and onlays, partial and full crowns, and three-unit fixed partial dentures in the anterior, premolar and posterior molar regions [12, 13].

When studying the clinical outcome of all-ceramic restorations, it is important to remember that aging and stress exhaustion in the oral environment, as well as function and para-function, have an impact on their longevity [14]. As a result, the gold standard in the dentistry literature is an evaluation that takes into account at least 5 years of clinical treatment [15, 16].

Only a few researches have published long-term clinical results. In general, clinical investigations demonstrate that crowns put in the posterior region have a higher fracture rate [17, 18], and molar crowns have a higher failure rate than premolar crowns [19].

Therefore, the aim of this study was to evaluate the success rate of chair-side fabrication of inlays or onlays, veneers (Vs), single crowns (SCs), and fixed partial dentures (FPDs) from lithium disilicate for up to 5 years. The null hypothesis was that four types of lithium disilicate ceramic restoration exhibit a similar clinical performance up to 5 years of clinical use.

## Materials and methods

### Study design

A total of 87,203 ceramic restorations were classified into four different categories comprising 2007 (2.3%) inlays or onlays, 66,637 (76.52%) Vs, 12,404 (14.22%) SCs, and 6060 (6.9%) FPDs respectively. This retrospective observational study was reviewed and approved by the Ethical Committee of the College of Dentistry of the University of Sulaimani (application no. 200).

All restorations were made of IPS e.Max lithium disilicate CAD/CAM ceramic blocks (Ivoclar-Vivadent, Liechtenstein), fabricated using software CEREC Inlab SW 4.2.4 CAD/CAM system (Sirona Dental Systems, Bensheim, Germany), and the final design was sent to the milling unit at inLab MC XL CEREC (Sirona Dental Systems GmbH, Bensheim, Germany). These large datasets were assembled by sixteen dentists over a period of 5 years and were recovered from the databases of four CAD/CAM machines. The database consisting of all types of restorations and complaint information within the time span from March 2015 to June 2020 (5-y interval) was released by the private B&R Dental Center to the authors.

### Patient selection

Patients presenting with dental defects including short clinical crowns, fractured restorations, poor spacing, misalignment or tooth discoloration were included for all-ceramic restorations, while those with poor oral hygiene, high caries activity or inadequate endodontic therapy were excluded.

The inclusion criteria for the abutment teeth were as follows: tooth mobility ≤ grade 1, vital pulp or successful endodontic treatment without apical periodontitis, no internal or external root resorption and normal occlusion relationship. Cantilever and long span FDPs with more than three units were excluded. Inlay or onlay ceramic restorations in the posterior premolar and molar regions were included, as were SCs and FDPs in the anterior and posterior regions and Vs in the anterior and premolar regions [13].

In the final patient sample, the mean age was 43.7 years, with a minimum age of 20.0 years and maximum of 74.0 years. The gender distribution was 57.46% females and 42.54% males.

### Prosthodontic procedures

#### Tooth preparation

Rubber dam was used to isolate the moisture and the abutment teeth were conventionally prepared according to the clinically standardized method. The crowns, veneers, inlay and onlay ceramic restorations were prepared according to the accepted principles [20–22]. Cavity walls were flared 6°–12°, isthmus minimum 1.5 mm width, internal lines and point angles rounded, pulpal floor shaped to allow an occlusal thickness of the indirect restorations of at least 1.5–2.0 mm and non-working and working cusps covered with at least 1.5 mm and 2 mm of restorative material, respectively. The minimum thicknesses of crowns preparation according to the study protocol in various areas are 2 mm on the occlusal or incisal surfaces, 1.5 mm on the labial or buccal surfaces, 1 mm on the proximal and lingual surfaces and 1 mm at a distinct chamfer of 1.0 mm width as finish line, respectively. The minimum thicknesses of veneers preparation in various areas are 0.3 mm at the cervical 1/3, 0.5 mm at the middle 1/3, 0.8 mm at the incisal 1/3 of the labial surfaces and 0.3 mm at a distinct chamfer of 1.0 mm width as finish line, respectively [13, 14]. If the preparation margin was located subgingivally, retraction cords were inserted, so that the finish line was clearly visible.

#### Scanning and restoration fabrication

In the private B&R Dental Center, the optical impressions were taken with an infrared camera, and the restorations were fabricated using the CEREC Inlab SW 4.2.4 CAD/CAM software system. All the restorations were made with IPS e.Max lithium disilicate CAD (Ivoclar Vivadent, Switzerland). At regular milling speed, the clinician milled restorations from prefabricated block of IPS e.max CAD. After removing the restoration from the milling chamber, the clinician cleaned and dried it carefully before using e.max CAD Crystall./Glaze paste (Ivoclar Vivadent) with shade tints to match the shade of the existing teeth. To complete the crystallization process, the restoration was burned in a porcelain oven under vacuum, according to the manufacturer's instructions. The fire cycle comprised two steps that took 35 min to complete (though this time has subsequently been cut in half thanks to the introduction of a spray glaze).

#### Restoration cementation

The intaglio surface of the restorations was etched with hydrofluoric acid (IPS Empress etch, Ivoclar Vivadent) for 20 s before adhesive cementation, and a silane coupling agent was used for 60 s (Monobond S, Ivoclar Vivadent). Using pumice and hand equipment, the tooth surface was mechanically cleansed. A dual cure self-adhesive resin cement was used to adhere the repairs (Multilink Sprint, Ivoclar Vivadent). After complete seating, the restoration margins were light cured for 3 s to remove excess cement and finally light cured for 20 s.

### Clinical evaluation

Data collection was performed through registration of the records of all patients from the database in the private B&R Dental Centre for up to 5 years. Each patient regularly follows up every 6 months and also any patient who visited for any complaint before follow up time would also be recorded. Within this retrospective study, the failures were reported using modified criteria developed by the US Public Health Service (USPHS) [23]. Failures were defined by any biological complication marginal adaptation, endodontic intervention, secondary caries, and periodontal problem or esthetic failure like color match and mechanical (technical) complication like clinical unacceptable fracture or chipping of ceramic and debonding (loss of retention). In the present study, the most common causes of fracture were evaluated and classified into span length, connector, occlusion, FPDs including molar, and others. Also, failure distribution was classified according to gender, arch (maxilla and mandible), and tooth position (anterior, premolar, and molar region). The failure rate was assessed as the number of failure restorations / the number of total restorations up to 5 years. The dentists who accomplished the recall examinations and follow ups to record the complications were the same ones who conducted the treatment. The authors were fully blinded to patients and dental practices and processed the database by filtering the information corresponding only to the lithium disilicate ceramic restorations.

### Statistical evaluation

SPSS version 22 was used to conduct statistical analysis (IBM, Armonk, NY, USA). Kaplan–Meier probabilities of time-dependent time-to-failure/complication were estimated based on the number of failures documented throughout the observation period for all types of ceramic restorations. The aforementioned parameters were analyzed by log-rank testing for significant associations with restorative failure. The Chi-squared test was used to assess the variations in causes of failure rates among the various restorations. Statistical analyses were conducted at a significance level of 0.05.

## Results

**Fig. 1:**
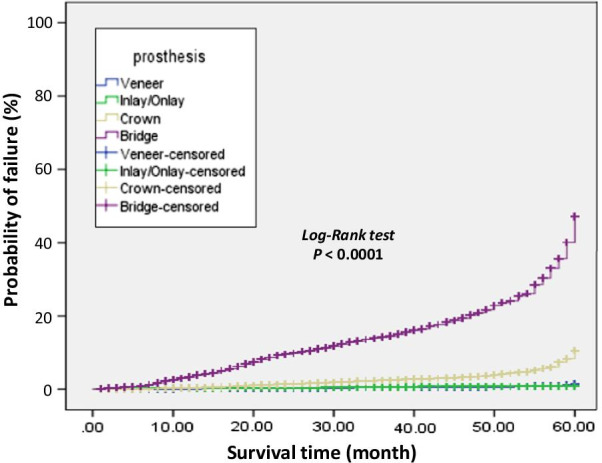
Kaplan–Meier one minus survival plot showing the cumulative probability of failure of lithium disilicate ceramic restorations after an observation period of up to 5 years. The P value refers to a comparison between the four restoration types using the log-rank test

The results from the Kaplan–Meier survival analysis are summarized in Table 1 and cumulative probability of failure illustrated in Fig. 1. Different types of prosthesis showed significant differences in survival time using log-rank test (P < 0.0001). At 5 years (60 months) follow up, inlay/onlay and Vs showed the highest cumulative survival probability (99.4%, 98.6%) and mean of survival (59.707, 59.763 months, respectively). In SCs, the cumulative survival probability was 89.5%, followed by FPDs which had the least cumulative survival probability (52.9%) and lowest mean of survival (52.373 months).

**Table 1:** Five-years Kaplan–Meier survival analysis

Prosthesis	Total number	Event (failure)		Censored		Cumulative survival probability (percent)	Estimate mean of survival (month)	95% confidence interval		Log-Rank (Mantel–Cox)
		Number	Percent	Number	Percent			Lower bound	Upper bound	
Veneer	66,732	234	0.35%	66,498	99.65%	98.6%	59.763	59.732	59.794	X^2^ = 5652.3 P < 0.0001
Inlay/onlay	2007	9	0.45%	1998	99.55%	99.3%	59.707	59.514	59.900	
Single crown	12,404	228	1.8%	12,176	98.2%	89.5%	58.722	58.556	58.888	
Fixed partial denture	6060	736	12.1%	5324	87.9%	52.9%	52.373	51.875	52.871	
Total	87,203	1207	0.6%	85,996	98.6%	85.08%	59.049	58.996	59.103

To compare the causes of failure in relation to different factors, out of 1207 overall failures, only 503 cases were analyzed, whereas 704 cases (569 units of FPD, 39 crown, 1 inlay and 95 veneers) were excluded from the study because the causes of failure were unknown (not recorded). Regarding FPDs, the number of prostheses was used rather than the number of units.

Pearson’s chi-squared test indicated a significant relationship between type of ceramic restoration and cause of failure (P = 0.000). Fracture most frequently occurred in FPDs, followed by SCs (27.63 and 26.64 respectively). Debonding and endodontic intervention were highest in the Vs (12.72 and 2.19 respectively), while marginal adaptation was found more frequently in SCs and Vs (7.36 and 2.90, respectively) Table 2.

**Table 2:** Causes of failures of different types of ceramic restorations

Causes of failure	Ceramic restorations				Total	P value
	Veneers	Inlay/onlay	Single crowns	Fixed partial dentures		
Fracture	29 (5.77)	6 (1.19)	134 (26.64)	139 (27.63)	308 (61.23)	0.000
Debonding	64 (12.72)	0	5 (0.99)	0	69 (13.72)	
Marginal adaptation	15 (2.9)	1 (0.198)	37 (7.36)	8 (1.59)	61 (12.13)	
Color match	12 (2.39)	0	5 (0.99)	10 (1.99)	27 (5.37)	
Endodontic intervention	11 (2.19)	0	0	0	11 (2.19)	
Periodontal disease	2 (0.398)	0	3 (0.596)	3 (0.596)	8 (1.59)	
Secondary caries	6 (1.19)	1 (0.199)	5 (0.99)	7 (1.39)	19 (3.78)	
Total	139 (27.63)	8 (1.59)	189 (37.57)	167 (33.2)	503 (100)	

**Fig. 2:**
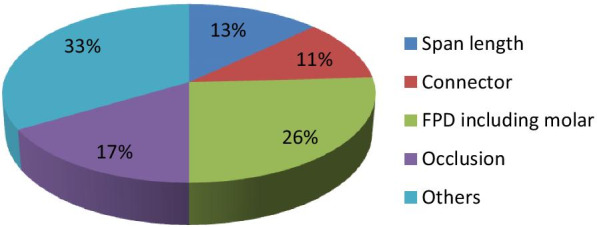
Common causes of fractures in ceramic FDP restorations

Among the most common causes of fracture in FPDs were short clinical crown, inadequate preparation of the abutment, improper design, and not following guidelines, which were categorized as others (33%), while inclusion of molar as abutment (26%) was indicated as the most likely individual cause of failure related to FPDs, as shown in Fig. 2.

Pearson’s chi-squared test identified a statistically significant association (P = 0.021) between causes of failure of ceramic restorations and dental arch. With the exception of periodontal disease, all the common causes of failure occurred more frequently in the maxillary arch compared to mandibular arch, as shown in Table 3.

**Table 3:** Frequencies of common causes of failure of ceramic restorations in the maxillary and mandibular arches

Causes of failure	Arch		Total	P value
	Maxillae	Mandible		
Fracture	188 (37.38)	120 (23.86)	308 (61.23)	0.021
Debonding	46 (9.15)	23 (4.57)	69 (13.72)	
Marginal adaptation	41 (8.15)	20 (3.98)	61 (12.13)	
Color match	24 (4.77)	3 (0.596)	27 (5.37)	
Endodontic intervention	10 (1.99)	1 (0.199)	11 (2.19)	
Periodontal disease	3 (0.596)	5 (0.99)	8 (1.59)	
Secondary caries	11 (2.19)	8 (1.59)	19 (3.78)	
Total	323 (64.21)	180 (35.79)	503 (100)	

Additionally, a significant relationship was found between frequency of cause of failure and position of the ceramic restoration in the dental arch (P = 0.000), using Pearson’s chi-squared test (p < 0.05). Fracture and marginal discrepancy occurred more frequently in the molar region (28.23% and 5.37%, respectively). Color match, debonding and endodontic intervention occurred most frequently in the anterior region, followed by the premolar region, and least frequently in the molar region, as shown in Table 4.

**Table 4:** Frequencies of common causes of failure of ceramic restorations according to tooth position (anterior, premolar, and molar)

Causes of failure	Tooth position			Total	P value
	Anterior	Premolar	Molar		
Fracture	95 (18.89)	71 (14.12)	142 (28.23)	308 (61.23)	0.000
Debonding	41 (8.15)	27 (5.37)	1 (0.199)	69 (13.72)	
Margin adaptation	18 (3.58)	16 (3.18)	27 (5.37)	61 (12.13)	
Color match	16 (3.18)	10 (1.99)	1 (0.199)	27 (5.37)	
Endodontic intervention	8 (1.59)	3 (0.596)	0	11 (2.19)	
Periodontal disease	1 (0.199)	3 (0.596)	4 (0.795)	8 (1.59)	
Secondary caries	7 (1.39)	4 (0.795)	8 (1.59)	19 (3.78)	
Total	186 (36.98)	134 (26.64)	183 (36.38)	503 (100)	

However, using Pearson’s chi-squared (P > 0.05), no significant relationship was identified between gender and different causes of failure of ceramic restorations (P = 0.3), as shown in Table 5.

**Table 5:** Frequencies of common causes of failures of ceramic restorations according to gender

Causes of failure	Gender		Total	P value
	Male	Female		
Fracture	135 (26.84)	173 (34.39)	308 (61.23)	0.3
Debonding	30 (5.96)	39 (7.75)	69 (13.72)	
Marginal adaptation	31 (6.16)	30 (5.96)	61 (12.13)	
Color match	4 (0.795)	23 (4.57)	27 (5.37)	
Endodontic intervention	6 (1.19)	5 (0.99)	11 (2.19)	
Periodontal disease	3 (0.596)	5 (0.99)	8 (1.59)	
Secondary caries	5 (0.99)	14 (2.78)	19 (3.78)	
Total	214 (42.54)	289 (57.46)	503 (100)	

## Discussion

There are few clinical studies that have compared the durability of various types of lithium disilicate ceramic restorations. The purpose of this study was to investigate the clinical success rate of lithium disilicate ceramic inlay or onlay, SCs, Vs, and FPDs restorations during a 5-year period. Besides the small methodological differences from the study by Belli et al. [24], including sample size, lifetime estimation and types of ceramic restorations, this study could be considered the first study to evaluate the CSR of four different types of ceramic restorations over a 5-year period or the most common causes of their failure in relation to arch, tooth position and gender.

In this study, the 5-year cumulative survival probability of the four different types of ceramic restorations, fabricated with lithium disilicate, was 85.08%. Inlay/onlay and Vs ceramic restorations survived longer compared to both SCs and FPDs, while FPDs showed significantly higher failure rates over 5 years. The results of the present study are comparable with some other studies. Gehrt et al. [25] reported that lithium disilicate crowns showed a sufficient survival rate (97.6%), while Sulaiman et al. [26], over a 45-month period, found only a 0.91% clinical failure rate for monolithic single unit IPS e.Max crowns and 1.83% failure for layered single unit crowns, with a combined failure rate of 1.15%. Additionally, Rauch et al. [21] reported that after 6 years, 87.6% of monolithic single unit crowns remained clinically acceptable, 70.1% without any complications. When compared to crowns with a zirconia substructure, Belli et al. [24] found that inlays and onlays would have a much longer predicted lifespan but a significantly lower estimated lifetime (10% failure in 30 years).

In the present study, it was reported that fracture was the main reason for failure of SCs and FPDs ceramic restorations, and this finding agrees with previous studies [27–30]. These failures may be attributed to psychological discomfort, underpreparation, not following the material guidelines regarding span length, tooth position, and inadequate thickness to withstand the occlusal load in posterior areas.

SCs and FPDs appeared to be strongly linked with the incidence of crown fracture in the current investigation, which was more common in the posterior locations. In the literature [27, 31, 32] this is also a common finding. This may be related to the lithium disilicate structure itself. It is prone to fatigue failure in clinical use due to its inherent brittleness and microcracks typically begin at load-bearing and/or stress-concentration sites, eventually fusing under dynamic loads and forming large faults that can weaken the lithium disilicate structure and lead to fracture [30, 33].

Marginal discrepancy was identified as one of the major problems associated with the failure of SCs and Vs fabricated with lithium disilicate, particularly in the posterior molar region. In the present study, complications due to inadequate marginal adaptation amounted to (12.13) and this could be attributed to difficulties in tooth preparation and scanning.

In the current study, clinical failure related to mismatch in color was more frequently present in the maxillary anterior region with Vs restorations, especially in females, and this was mainly due to teeth type and position and females’ higher esthetic demands. The findings are similar to those of Fasbinder et al. [34], who showed that after 2 years, 87.0% of IPS e.Max CAD SCs cemented with dual-cure self-etching cement (Multilink Automix [MA], Ivoclar Vivadent) had minor staining. Previous research [35, 36] found that wear of the luting material, as well as patient-related aspects like nutrition, smoking habits, and dental hygiene, all contribute to discoloration.

A limitation of the present retrospective study was that the dentists who performed the treatment also had to perform the recall examinations. Another shortcoming of the study was that include the fact that lithium disilicate was not highly recommended in FPDs in the posterior region due to its mechanical properties. Strengths of this investigation are the use of unique long-term data, large sample size, and the retrospective assessment of four different types of ceramic restorations made of lithium disilicate.

In conclusion, the data indicated that lithium disilicate ceramic restorations exhibited ideal medium term survival over all confounding variables studied. FPDs recorded the lowest survival rate and fracture was the most common cause of failure in both SC and FPD ceramic restorations.

## Data Availability

The datasets used and/or analyzed during the current study are available from the corresponding author on reasonable request.

## Abbreviations

Vs: Veneers
SCs: Single crowns
FPDs: Fixed partial dentures
